# Analysis of the *sericin*1 promoter and assisted detection of exogenous gene expression efficiency in the silkworm *Bombyx mori* L.

**DOI:** 10.1038/srep08301

**Published:** 2015-02-06

**Authors:** Lupeng Ye, Qiujie Qian, Yuyu Zhang, Zhengying You, Jiaqian Che, Jia Song, Boxiong Zhong

**Affiliations:** 1 College of Animal Sciences, Zhejiang University, Hangzhou 310058, P. R. China

## Abstract

In genetics, the promoter is one of the most important regulatory elements controlling the spatiotemporal expression of a target gene. However, most studies have focused on core or proximal promoter regions, and information on regions that are more distant from the 5′-flanking region of the proximal promoter is often lacking. Here, approximately 4-kb of the *sericin*1 (*Ser*1) promoter was predicted to contain many potential transcriptional factor binding sites (TFBSs). Transgenic experiments have revealed that more TFBSs included in the promoter improved gene transcription. However, multi-copy proximal *Ser*1 promoter combinations did not improve gene expression at the transcriptional level. Instead, increasing the promoter copy number repressed transcription. Furthermore, a correlation analysis between two contiguous genes, *firefly luciferase* (*FLuc*) and *EGFP*, was conducted at the transcriptional level; a significant correlation was obtained regardless of the insertion site. The ELISA results also revealed a significant correlation between the transcriptional and translational *EGFP* levels. Therefore, the exogenous gene expression level can be predicted by simply detecting an adjacent *EGFP*. In conclusion, our results provide important insights for further investigations into the molecular mechanisms underlying promoter function. Additionally, a new approach was developed to quickly screen transgenic strains that highly express exogenous genes.

The development of transgenic technology constituted a crucial milestone in the history of life science, and is frequently used to alter biological traits and produce valuable foreign proteins in various organisms. Gene transcription is regulated by multiple *cis*-acting elements, such as promoters, enhancers, insulators, and silencers^1^,^2^,^3^. The promoter is the closest and most important element regulating target gene transcription. Universally, the promoter is a region of essentially contiguous DNA sequence in the immediate vicinity of the transcription start site (TSS), which initiates the transcription of a specific gene. However, the transcription of diverse genes uses distinct promoter types^4^, and there is no universal promoter that suits all types of genes. The core promoter^5^,^6^, the most important part of an active promoter, is a short sequence around the TSS. It acts as a platform for the assembly of transcription pre-initiation complexes (PICs) that include RNA polymerase II (pol II) and certain basal or general transcription factors (TFs), such as TFIIA, TFIIB, TFIID, TFIIE, TFIIF, and TFIIH^7^,^8^. Eventually, pol II accurately and efficiently directs transcription initiation^9^,^10^. The reason for the anchoring of PICs onto the core promoter is the existence of several core promoter elements, such as the classical TATA box^11^, the Initiator (Inr)^12^,^13^,^14^, and downstream core promoter element (DPE)^15^. In the current study, proximal promoters were selected to drive target gene transcription *in vivo* or *in vitro*. The proximal promoter controls gene transcription even though it may not include all regulatory elements^4^. Nevertheless, regulatory elements distant from the proximal promoters may be necessary to enhance the promoter's strength. We speculated that appropriately extending the length of the 5′-flanking sequence of a proximal promoter may improve gene transcription efficiency. We also speculated that combining multi-copy promoters may similarly contribute to enriching the PICs and enhance transcription efficiency.

Foreign genes integrate into the organism's genome and induce position effects. These events are influenced by the surrounding host chromatin and result in an exogenous gene that will demonstrate dramatically different expression patterns in various insertion sites^16^,^17^,^18^,^19^,^20^. In most transgenic studies, exogenous genes often randomly integrate into a host genome, and only a very low proportion of the integration events occur at favorable positions^21^. Therefore, the expression levels of foreign genes in different integration sites need to be assessed to identify the organisms with high foreign gene expression level potential, particularly in development of bioreactors to produce as much foreign protein as possible. This type of assessment is a time-consuming and labor-intensive process. A marker gene is generally used in transgenic experiments to screen positive individuals. Therefore, we reasoned that if the foreign- and marker genes are adjacent and in the same transcriptional direction in transgenic vectors, they may be similarly affected by host chromatin. As a result, there should be a significant linear correlation between the expression levels of these two genes, even in different insertion sites. Therefore, a promising method for predicting foreign gene expression levels may be detection of an adjacent marker gene, which can be more easily conducted.

The domestic silkworm, *Bombyx mori*, is an important economic insect that can synthesize and spin a large variety of proteins in silk glands to produce a cocoon before pupating. A method based on a *piggyBac* (*PB*) transposon-derived vector was successfully developed for stable germline transformation in silkworms^22^. Since its development, several studies have been performed using silk gland specific expression promoters, such as *sericin* (*Ser*) and *fibroin* *light-chain* (*Fib-L*), to drive exogenous gene expression in silk glands. These ideal bioreactors have helped express many valuable foreign proteins. However, most studies have indicated that foreign protein expression is still very low and accounts for less than 1% of a cocoon's weight^23^,^24^,^25^,^26^,^27^,^28^,^29^,^30^. It is noteworthy that the *Ser* and *Fib-L* promoters used in these studies are proximal or shorter promoters. Furthermore, positive individuals have to be selected from a large number of treated silkworms, rendering the process tedious and time-consuming. It is therefore necessary to develop a more efficient method for transgenic organism screening.

The objective of this study was to assess the correlations between different lengths and copy numbers of the *Ser*1 promoter and the foreign gene transcriptional level in silkworms. Additionally, a correlation analysis between two adjacent genes, a marker gene (*EGFP*) and foreign gene (*FLuc*), was conducted to elucidate their relationship.

## Results

### Architectural analysis of *Ser*1 promoter

**Figure 1:**
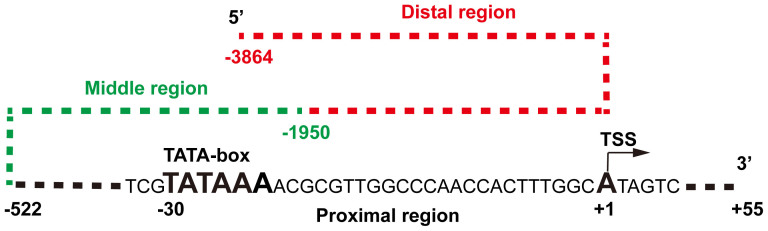
Schematic diagram of the *Ser*1 promoter sequence. Black dashed line and capital letters represent the proximal promoter region; green and red dashed lines represent the middle and distal promoter regions respectively. TSS, transcription start site.

Promoters can be divided into two classes, conserved TATA box-enriched promoters and evolvable CpG-rich promoters^4^. The 4-kb long 5′-flanking *Ser*1 gene sequence, a highly tissue-specific gene that is only expressed in the middle silk gland^31^, was cloned and analyzed using bioinformatics methods. We confirmed the positions of the *Ser*1 gene TSS and the classical regulatory element TATA-box, which was located 30 bp upstream of the TSS (Fig. 1). In addition, we also predicted the potential TFBSs in three *Ser*1 promoter regions. The results suggested that the promoter's proximal, middle, and distal regions comprised 41, 87, and 105 potential TFBSs, respectively (Supplementary Table S1). Thus, we speculated that the 192 potential TFBSs located further away from the proximal promoter region may be beneficial for downstream gene transcription.

### *Ser*1 promoter activity analysis

**Figure 2:**
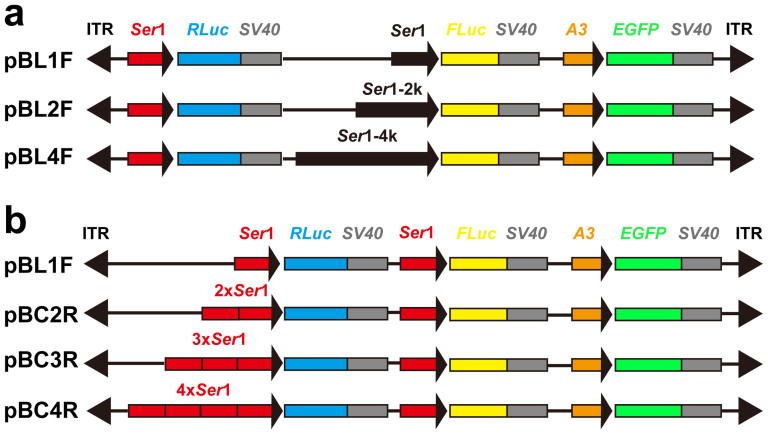
Structure diagram of transgenic vectors. *Ser*1, *Ser*1-2 k, and *Ser*1-4 k indicate the ~0.5 kb, ~2 kb and ~4 kb of *Ser*1 gene 5′-flanking sequence, respectively. The 2×, 3× and 4× *Ser*1 represent 2, 3 and 4 copies of *Ser*1 proximal promoter, respectively. *RLuc*, *renilla luciferase*; *FLuc*, *firefly luciferase*; A3, *Bombyx mori* A3 cytoplasmic actin gene promoter; *EGFP*, enhanced green fluorescence protein; *SV40*, 3′-untranslated sequences; ITR, inverted terminal repeats of *PB* transposon.

**Figure 3:**
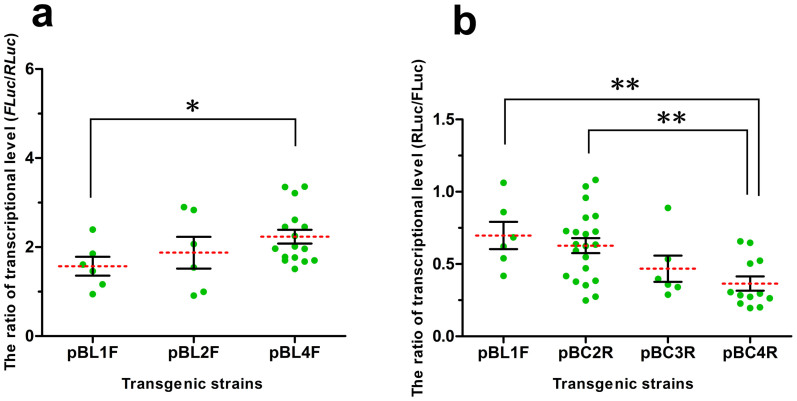
Comparison of driving activities of the *Ser*1 promoters. (a) Differences in *FLuc* transcription driven by different lengths of the *Ser*1 promoter. The final relative transcriptional level was calculated as *FLuc* relative expression level/*RLuc* relative expression level. (b) Differential transcription of *RLuc* driven by different copy numbers of proximal *Ser*1 promoter. Similarly, the final relative expression level was calculated as *RLuc* relative expression level/*FLuc* relative transcriptional level. *P < 0.05, **P < 0.01.

To accurately distinguish the different lengths of the *Ser*1 promoter that drive activity while eliminating the position effects as much as possible, we designed two expression frames in the same direction that were closely inserted in vectors (Fig. 2a). A reference gene (*renilla luciferase, RLuc*), driven by a proximal *Ser*1 promoter, was used in each vector. The ratio of *FLuc*/*RLuc* gene expression at the transcriptional level was calculated to determine the strength of the three different *Ser*1 promoters according to their lengths. Three types of pBL series-positive transgenic silkworms were obtained (Supplementary Table S2). Certain positive broods included multiple phenotypes, which mainly manifested in diverse patterns of *EGFP* expression resulting from *PB* integrating into different sites. This outcome is a common phenomenon in *PB*-mediated gene transposition studies. Therefore, silkworms with different *EGFP* expression patterns in the same brood were separated and defined as independent transgenic strains. We found that the highest level of *FLuc* transcription was in the pBL4F series transgenic silkworms, followed by the pBL2F series (Fig. 3a). To our surprise, the strength comparison among the four types of proximal promoter combinations (Fig. 2b) revealed that multi-copy proximal promoters did not enhance *RLuc* (*FLuc* served as an internal reference gene) transcription but repressed transcription (Fig. 3b). Indeed, *RLuc* transcription decreased as the promoter copy number increased.

### Integration site analysis in transgenic silkworms

**Figure 4:**
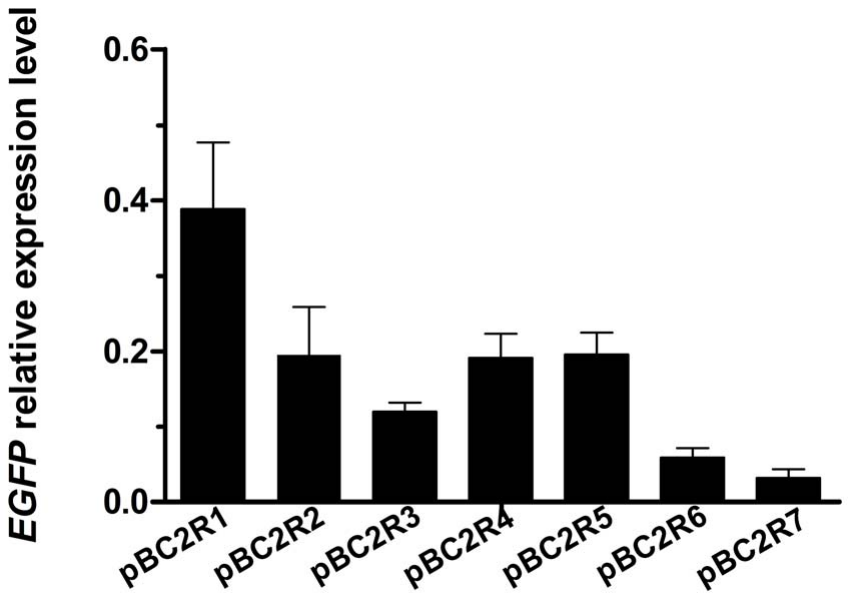
Transcriptional level of *EGFP* in different integration sites. The inverse PCR showed that the *PB* integration sites were different in the seven pBC2R transgenic strains (Supplementary Table S3). The *EGFP* mRNA levels differed among these strains due to position effects from the host chromatin. Data are presented as the mean ± SD of three separate experiments performed in triplicate.

A foreign gene in different genomic loci demonstrates diverse expression levels due to position effects. An integration site analysis was conducted in the pBC2R series transgenic strains, which yielded the most positive individuals. As a representative sample, we selected seven transgenic strains, and each was found to have *PB* inserted at different genome positions (Supplementary Table S3). Meanwhile, the *EGFP* transcription levels were also determined in these strains, and huge differences were found among these levels; specifically, the transcription level of *EGFP* in pBC2R1 was 12 times higher than that in pBC2R7 (Fig. 4). These results further confirmed that *PB*-mediated transgenesis resulted in random insertions into the genome and that foreign gene transcription is dramatically influenced by the position effect.

### Correlation analysis between marker- and target genes

**Figure 5:**
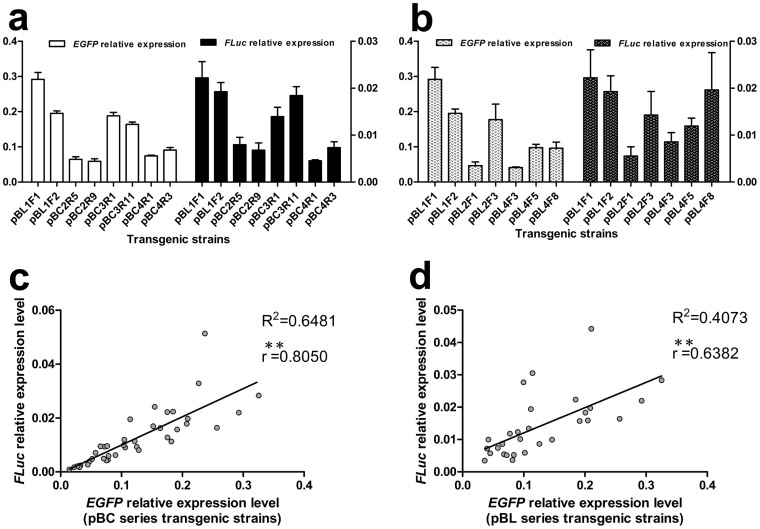
Correlation analysis between the marker (*EGFP*) and target (*FLuc*) genes at the transcriptional level. (a, c) Transcription pattern comparison of *EGFP* and *FLuc* in the pBC (including pBL1F) series transgenic silkworms. A significant correlation was revealed between the *EGFP* and *FLuc* expression levels with the two expression frames assembled in close proximity. (b, d) However, the correlation between *EGFP* and *FLuc* in the pBL series transgenic silkworms was relatively lower; the *Ser* 1 promoters in front of *FLuc* were different (Fig. 2a), which affected the transcriptional level of *FLuc*. The qRT-PCR data are shown as the mean ± SD of three separate experiments performed in triplicate. **P < 0.01.

Two adjacent foreign genes should theoretically experience similar effects from the host chromatin. In our study, the marker gene was inserted close to the target gene with the same transcriptional orientation, e.g., *EGFP* and *FLuc* in Fig. 2. We speculated that the transcriptional levels of these two adjacent genes would demonstrate a linear correlation even if they were inserted into different genomic loci. Four series of transgenic silkworms, pBL1F, pBC2R, pBC3R, and pBC4R, shared a common feature, the promoter in front of *FLuc* and *EGFP* was unchanged (Fig. 2b). The qRT-PCR results indicated that the *FLuc* and *EGFP* transcriptional levels varied for the different integration sites, but the two adjacent genes exhibited similar expression patterns (Fig. 5a) with a significant correlation between their expression levels (Fig. 5c). However, the correlation among the pBL1F, pBL2F, and pBL4F series transgenic silkworms was lower compared to the previous four series of transgenic silkworms (Fig. 5b, d). This finding may be due to the different lengths of the *Ser*1 promoter in front of *FLuc*, which resulted in different *Ser*1 promoter strengths.

**Figure 6:**
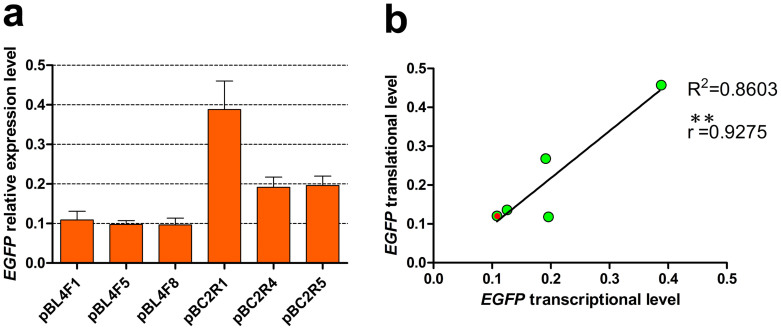
Correlation analysis between the *EGFP* transcriptional and translational levels. (a) Six strains with different *EGFP* transcription levels were selected for further analysis. Data are presented as the mean ± SD of three separate experiments performed in triplicate. (b) There was a highly significant correlation between the transcriptional and translational levels of *EGFP*. Each green dot represents one transgenic strain; a red dot is used to distinguish two overlapping dots. **P < 0.01.

These findings suggested that *FLuc* expression was significantly correlated with *EGFP* expression at the transcriptional level. To determine whether the transcriptional and translational levels were also significantly correlated at the same developmental stage, we selected three strains with low and relatively high *EGFP* transcription from the pBL4F and pBC2R series transgenic silkworms, respectively (Fig. 6a). The ELISA results suggested that there was a highly significant correlation between the transcriptional and translational *EGFP* levels (Fig. 6b). Taken together, these results demonstrate that the expression level of a target gene (*FLuc*) in the host can be simply predicted by the expression level of an adjacent marker gene (*EGFP*).

## Discussion

It is important to develop high-efficiency bioreactors that meet societal demands. Identifying efficient *cis*-acting regulators, such as promoters and enhancers, is commonly used to improve foreign gene expression. Previous studies have demonstrated that TATA box-containing promoters are more constrained with slow evolution rates. The preferred distance between a TATA-box and TSS is 30 or 31 bp, and this promoter type generally has high tissue specificity in driving gene expression^4^. These findings are highly consistent with our results, and we further validated the characteristics of the TATA box-containing promoter. Promoter activity is closely related to its sequence length; longer promoters are superior regardless of the quantity and type of potential TFBSs (Supplementary Table S1). The 192 potential TFBSs in the middle and distal *Ser*1 promoter regions further enhanced promoter activity and facilitated downstream gene transcription. Therefore, we believe that the TFBSs are important parameters for improving transgene expression efficiency. However, lengthening the upstream regions of the *Ser*1 promoter did not demonstrate a pronounced improvement in the promoter activity; the promoter activity improved by approximately 1.5 times after using a 4-kb length *Ser*1 promoter. Currently, using efficient enhancers is a common method for enhancing the efficiency of exogenous gene expression in silkworms. However, expression efficiency remains very low. We believe that the precondition for improving transgene expression efficiency is to find a high-efficiency promoter. Therefore, promoter activity will be significantly amplified after the combined use of enhancers, and using the intrinsic functional elements of the promoter as much as possible is an efficient method for improving the level of exogenous gene expression.

**Figure 7:**
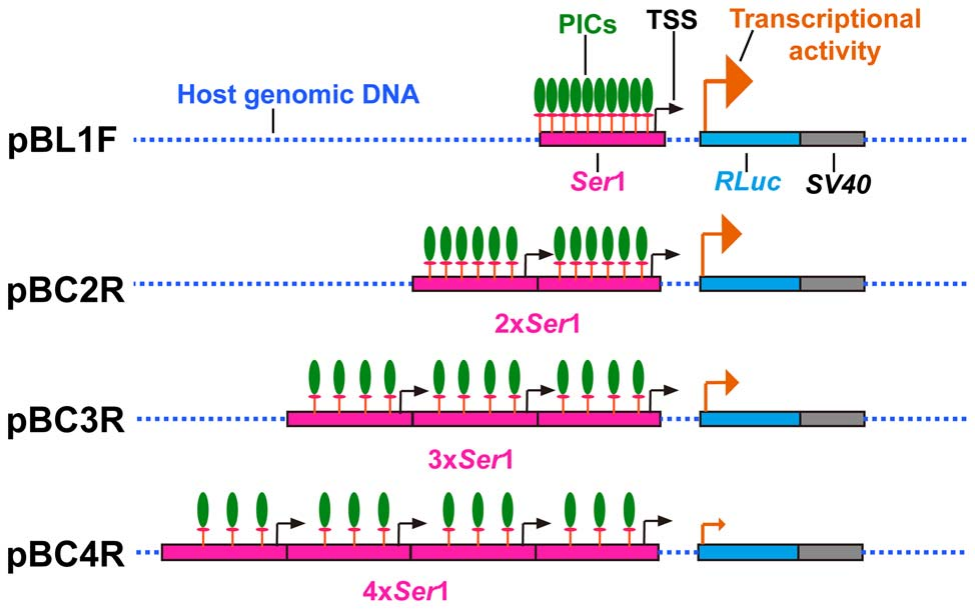
Schematic representation of the competition phenomenon among multi-copy promoters. PICs, transcription pre-initiation complexes; TSS, transcription start site of the *Ser*1 promoter; orange arrows represent transcription activity and transcription orientation of *RLuc*; 2×, 3× and 4× of *Ser*1 represent 2, 3 and 4 copies of the *Ser*1 proximal promoter, respectively; *RLuc*, *renilla luciferase*; *SV40*, 3′-untranslated sequences.

Surprisingly, the multi-copy promoter combinations had an adverse effect on gene transcription. The activity of weakened multi-copy promoter combinations may generate competition among the same promoters. This possibility is reflected in the simultaneous competition for the same TFs and pol II. We speculate that this phenomenon will induce limited substrates that dispersedly combine on promoters, with the promoter closest to the downstream gene serving as the main driving force of gene transcription (Fig. 7). As a result, the promoters function at a sub-optimal level due to a lack of PICs (composed of TFs and pol II) on the promoter closest to the gene. In addition, each proximal promoter contains a TSS (Fig. 7), implying another possibility that competition also exists among multiple TSSs. However, more detailed studies are necessary to investigate these two possible molecular mechanisms.

Transgenic vectors often contain two parts, the marker gene and the exogenous gene expression cassettes. Our design approach was different from the standard approach, which does not take into consideration the special demands of transgenic vector construction. The marker gene has only been used to select transgenic animals in previous studies. However, another important function of the marker gene is to assist in determining the exogenous gene expression level, as described above. Our findings revealed that if the two expression cassettes are constructed close to one another with the same transcriptional orientation, then there is a significant linear correlation between their transcriptional levels, even if they are in different insertion sites. Furthermore, the transcriptional and translational levels of the marker genes were significantly correlated; therefore, it is possible to determine foreign gene expression levels by simply assessing the level of marker gene expression. Marker genes are widely used in life science studies and can be easily and conveniently measured using various methods. In conclusion, the assisted assessment of the expression level of an exogenous gene is a promising new method for the large-scale screening of transgenic strains characterized by a high-expression capacity for foreign proteins. Additionally, it can also be used for the real-time monitoring of gene expression patterns to more conveniently and thoroughly understand gene functions. Furthermore, it provides a foundation for further optimizing transgenesis technology.

## Methods

### Silkworm strains

*Lan10*, the multivoltine with diapause ability silkworm strain, and *Qiufeng*, a high silk-yield strain, were used. All experimental silkworms were reared on fresh mulberry leaves under the standard conditions (25°C, 80% R.H).

### Bioinformatics analysis of *Ser*1 promoter

The preselected ~4-kb length 5′-flanking *Ser*1 gene sequence was analyzed using the TFSEARCH (version 1.3) software^32^ to identify putative TATA-box and TFBSs (threshold value: 85.0). Moreover, the TSS location was also analyzed using the Neural Network Promoter Prediction software^33^.

### Transgenic vector construction

Genomic DNA was extracted from *Qiufeng* silk glands using a DNA extraction kit (Sangon, Shanghai, China). Then, different *Ser*1 promoter lengths, 0.5 kb (proximal region, 577 bp), 2 kb (proximal and middle region, 2005 bp) and 4 kb (proximal, middle, and distal region, 3919 bp), were cloned using high fidelity PrimeSTAR HS DNA Polymerase (TAKARA BIO INC., Otsu, Shiga, Japan). The promoters were sequenced after cloning into the pMD19-T vector (TAKARA BIO INC., Otsu, Shiga, Japan). Three transgenic vectors, pBL1F, pBL2F, and pBL4F, were constructed based on the bi-fluorescence plasmid pA3RLucFLuc (conserved in our lab) and the *PB* transposon plasmid pBA3EGFP to compare the driving activities of the three different *Ser*1 promoters. *EGFP* served as a marker gene used for positive individual screening, and *RLuc* was an internal reference gene to standardize the *FLuc* transcription level. Furthermore, combinations of two, three, and four copies of *Ser*1 proximal promoters were constructed in the pBC2R, pBC3R, and pBC4R vectors, respectively, and their driving activities were compared. Similarly, *FLuc* was used as an internal reference gene to standardize the *RLuc* transcription level. All data were analyzed by multiple comparisons (LSD) after one-way analysis of variance (ANOVA).

### Embryo microinjection and positive individual screening

The detailed methodologies for embryo microinjection and positive animal screening have been previously described in detail^22^,^34^. Briefly, the fertilized eggs were timely collected and microinjection was performed within 8 h of oviposition. The injection plasmids consisted of transposition- and helper plasmids, at a concentration ratio of 1:0.5, and a total injection volume of approximately 10 nL for each egg (the total concentration of DNA was 0.4 μg/μL) was used. After microinjection, G0 generation zygotes were cultured under standard conditions to mature moths. Each moth was mated with native *Lan10* silkworms to produce G1 generations. Finally, positive individuals were selected from each G1 brood using an Olympus SZX16 fluorescence microscope (Olympus, Tokyo, Japan).

### Total RNA Extraction and Quantitative RT-PCR Analysis

Certain procedures were performed as previously described^35^. Briefly, total RNA was extracted from fresh middle silk glands collected from day 3 fifth instar larvae using TRIzol reagent (Invitrogen, Carlsbad, CA, USA) according to the manufacturer's instructions. The cDNA was obtained using the PrimeScript RT reagent kit with gDNA Eraser (Perfect Real Time) (TAKARA BIO INC., Otsu, Shiga, Japan). The qRT-PCR was performed by a LightCycler480 instrument (Roche Diagnostics, Rotkreuz, Switzerland) in a 20-μL volume reaction that included 50 ng cDNA, 10 μL of SYBR *Premix Ex Taq*™ (2X) (TAKARA BIO INC., Otsu, Shiga, Japan), and 4 μM each of forward and reverse primer. The amplification program began with 30 s denaturation at 95°C, followed by 40 cycles of 5 s at 95°C, 20 s at 60°C, and 15 s at 72°C. The relative quantification analyses of the mRNA levels of target genes were based on the delta Ct value normalized with a reference gene, Rp49 gene (accession number: NM_001098282). Each sample had three independent replicates, and the gene-specific primers (Supplementary Table S4) were designed using the Primer Premier 5.0 software (Premier Biosoft International, Palo Alto, CA).

### Inverse PCR analysis

Genomic DNA was isolated from the positive transgenic silkworms as described above. The inverse PCR analysis was conducted as previously described^22^, 1 μg of each genomic DNA was digested with *Sau*3A I at 37°C for 2 h and then self-circularized overnight at 16°C using T4 DNA ligase (TAKARA BIO INC., Otsu, Shiga, Japan). The ligated products (approximately 50 ng DNA) were amplified using *EX* *Taq* polymerase (TAKARA BIO INC., Otsu, Shiga, Japan) and the designed primers (Supplementary Table S4) with 4 min denaturing cycle at 94°C, followed by 40 cycles of 30 s at 94°C, 30 s at 58°C, and 2 min at 72°C, and a final extension at 72°C for 10 min. Amplified products were sequenced after cloning into pMD19-T to determine the insertion sites.

### Enzyme-linked immunosorbent assay (ELISA)

The middle silk glands were dissected from the day 3 fifth instar larvae of transgenic silkworms and were thoroughly grinded in liquid nitrogen after adding ice-cold 2× cell lysis buffer (Cell Signaling Technology, Inc., Danvers, MA, USA) at a ratio of 100 mg of tissue to 400 μL buffer. Next, the samples were incubated on ice for 30 min with vortexing several times, centrifuged at 15,000 × *g* at 4°C for 10 min, and then the supernatant was collected in a new tube. The protein concentration was determined using a *DC* protein assay kit (Bio-Rad, California, USA). The tissue lysates were stored at −80°C in single-use aliquots. Each tissue lysate was diluted to the same concentration (10 mg/mL), and 1 mg of total protein was used for the ELISA assay. *EGFP* was detected using the PathScan® Total GFP Sandwich ELISA Kit (Cell Signaling Technology, Inc., Danvers, MA, USA) according to the manufacturer's instructions. Finally, the absorbance readings at 450 nm were measured using a microplate reader (PerkinElmer, Massachusetts, USA).

## Author Contributions

L.Y. designed and conducted experiments, performed data analysis and wrote the paper. Q.Q. conducted experiments and performed data analysis. Y.Z. and Z.Y. conducted experiments. J.C. and J.S. reared and harvested all the samples. B.Z. conceived, designed and conducted experiments, performed data analysis, and revised the manuscript. All authors read and revised manuscript before submission.

## Supplementary Material

Supplementary Information Supplementary information
