# Supplementary material for: Analysis of the sericin1 promoter and assisted detection of exogenous gene expression efficiency in the silkworm Bombyx mori L
Source: Sci Rep. 2015 Feb 6;5:8301. doi: 10.1038/srep08301 (PMC4319154; doi:10.1038/srep08301)
Supplement: Supplementary Information [file srep08301-s1.pdf]

# Analysis of the *sericin*1 promoter and assisted detection of exogenous gene expression efficiency in the silkworm *Bombyx mori* L.

Lupeng Ye[1], Qiujie Qian[1], Yuyu Zhang[1], Zhengying You[1], Jiaqian Che[1], Jia Song[1] & Boxiong Zhong[1]*

[1]College of Animal Sciences, Zhejiang University, Hangzhou 310058, P. R. China

*Corresponding author. Tel:/Fax: +86-571-86971302; E-mail: bxzhong@zju.edu.cn

**Table S1. Statistical analysis of TFBSs on the three regions of the *Ser*1 promoter**

| **Proximal region of the *Ser*1 promoter** | | **Middle region of the *Ser*1 promoter** | | **Distal region of the *Ser*1 promoter** | |
|---|---|---|---|---|---|
| Potential TFBSs | Number (total=41) | Potential TFBSs | Number (total=87) | Potential TFBSs | Number (total=105) |
| BR-C Z | 4 | BR-C Z | 2 | BR-C Z | 12 |
| Croc | 2 | Croc | 2 | HSF | 39 |
| HSF | 13 | HSF | 42 | Dfd | 18 |
| Dfd | 16 | Dfd | 16 | Ttk | 1 |
| Bcd | 1 | Bcd | 3 | Hb | 25 |
| Ttk | 1 | Ttk | 1 | dl | 3 |
| Hb | 4 | Hb | 10 | CF2-II | 5 |
| | | Ftz | 3 | GCM | 2 |
| | | Abd-B | 2 | | |
| | | dl | 1 | | |
| | | STAT | 2 | | |
| | | CF2-II | 2 | | |
| | | Su(H) | 1 | | |

**Table S2. Outcome of transgenesis**

| Transgenic strains | Microinjected eggs (G0) | Hatched eggs (Hatching rate, %) | G1 generation broods | *EGFP*-positive broods | Percentage of transgenic efficiency (%) |
|---|---|---|---|---|---|
| pBL1F | 1751 | 89 (5.1) | 47 | 8 | 17.0 |
| pBL2F | 2335 | 84 (3.6) | 60 | 8 | 13.3 |
| pBL4F | 672 | 22 (3.3) | 16 | 2 | 12.5 |
| pBC2R | 627 | 63 (10.0) | 50 | 8 | 16.0 |
| pBC3R | 1320 | 154 (11.7) | 21 | 5 | 23.8 |
| pBC4R | 950 | 96 (10.1) | 40 | 6 | 15.0 |

**Table S3. Integration sites of transgenic silkworms**

| Transgenic strains | Hit chromosome position | Hit scaffold position | Distribution of *piggyBac* insertions in genes | 5'-flanking genomic DNA sequence | Vector | 3'-flanking genomic DNA sequence |
|---|---|---|---|---|---|---|
| pBC2R1 | Chr. 16 | Bm_scaf 39 | Exon | TTTAGATCTACCATTA**TTAA** | *piggyBac* | **TTAA**AAGAGGCTGGCGACA |
| pBC2R2 | Chr. 21 | Bm_scaf 7 | Intergenic | TCTGTATTTGAGTTCA**TTAA** | *piggyBac* | **TTAA**GGGCTGGGCAAACGT |
| pBC2R3 | Chr. 25 | Bm_scaf 57 | Intergenic | CGAAATTATATACCTCA**TTAA** | *piggyBac* | **TTAA**TAAGCTCTAAATTTTA |
| pBC2R4 | Chr. 3 | Bm_scaf 17 | Intron | AATTAATACTTATTCA**TTAA** | *piggyBac* | **TTAA**TAAGTTTTACGAAGTT |
| pBC2R5 | Chr. 21 | Bm_scaf 7 | Intergenic | ATAAGTATATCGTTGC**TTAA** | *piggyBac* | **TTAA**TATAATGTTAACAAAG |
| pBC2R6 | Chr. 1 | Bm_scaf 8 | Intergenic | TTACTTGATTAATTCAC**TTAA** | *piggyBac* | **TTAA**CGGCGCTCATTACATA |
| pBC2R7 | Chr. 10 | Bm_scaf 253 | Exon | ACTTTCTGAAGGGTCT**TTAA** | *piggyBac* | **TTAA**ACAATGATTTCAATAA |

## Table S4. Primers for study

| Target genes | Forward primers (5’→3’) | Reverse primers (5’→3’) | Purpose |
|---|---|---|---|
| *Ser*1 promoter | GCACACACACTACATACCAT | GTTGGCGGTCTTTGGATCG | 0.5 kb length *Ser*1 promoter cloning |
| *Ser*1 promoter | ATGCTCGTCTGTCAAGGGTCT | GTTGGCGGTCTTTGGATCG | 2 kb length *Ser*1 promoter cloning |
| *Ser*1 promoter | CGGCTTCCAACGCCTTTGTTATT | GTTGGCGGTCTTTGGATCG | 4 kb length *Ser*1 promoter cloning |
| *FLuc* | TAAAGACTTCAAGCGGTCAACTATG | TTATGTCCGGTTATGTAAACAATCC | qRT-PCR |
| *RLuc* | TTGGCACCTTCAACAATAGCAT | GCCTCGTGAAATCCCGTTAGT | qRT-PCR |
| *EGFP* | AGCAGAAGAACGGCATCAAGGTG | CGCTTCTCGTTGGGGTCTTTG | qRT-PCR |
| *Rp*49 | TGCTCCCAAATGGATTCCGTAAG | CACGATCAGCTTCCGCTTCTTC | Reference gene for qRT-PCR |
| *piggyBac* left arm | CTTACCGCATTGACAAGCACGCC | GTTCTTTAGACGATGAGCATATCCT | Inverse PCR |
| *piggyBac* right arm | TATCTTTAACGTACGTCACAATATG | GGTCTGTATATCGAGGTTTATTTA | Inverse PCR |
